# Harnessing the microbiomes of *Brassica* vegetables for health issues

**DOI:** 10.1038/s41598-017-17949-z

**Published:** 2017-12-15

**Authors:** Birgit Wassermann, Daria Rybakova, Christina Müller, Gabriele Berg

**Affiliations:** Graz University of Technology, Institute of Environmental Biotechnology, Petersgasse 12, 8010 Graz, Austria

## Abstract

Plant health is strongly connected with plants´ microbiome. In case of raw-eaten plants, the microbiome can also affect human health. To study potential impacts on health issues of both hosts, the microbiome composition of seven different *Brassica* vegetables, originating from different food processing pathways, was analyzed by a combined approach of amplicon sequencing, metagenomic mining and cultivation. All *Brassica* vegetables harbored a highly diverse microbiota as identified by 16S rRNA gene amplicon sequencing. The composition of the microbiota was found to be rather driven by the plant genotype than by the processing pathway. We characterized isolates with potential cancer-preventing properties by tracing myrosinase activity as well as isolates with biological control activity towards plant pathogens. We identified a novel strain with myrosinase activity and we found bacterial myrosinase genes to be enriched in rhizosphere and phyllosphere metagenomes of *Brassica napus* and *Eruca sativa* in comparison to the surrounding soil. Strains which were able to suppress plant pathogens were isolated from naturally processed vegetables and represent a substantial part (4.1%) of all vegetable microbiomes. Our results shed first light on the microbiome of edible plants and open the door to harnessing the *Brassica* microbiome for plant disease resistance and human health.

## Introduction

The plant microbiome has been intensively studied for more than a century^1^ but recently developed omics technologies provide much deeper insights into the plant-associated microbial diversity^2,3^. Distinct microbiomes have been identified for each organ^4,5^, and the composition of bacterial communities in the rhizosphere is influenced by the plant genotype^6^. The enrichment of microorganisms by the plant root is not a random, but rather a targeted process: the current model shows the involvement of seed-borne microorganisms^7,8^, and the attraction of microbes from the soil^9^ in combination with plant-specific secondary metabolites acting as repellents^10^ and plant defense signaling^11^. Although the structure of plant microbiomes especially in the rhizosphere is well-studied, there are many knowledge gaps due to the plant-specific species component and because most of the studies were performed on crops and model plants like *Arabidopsis* ^12,13^. The importance of the plant microbiome as key determinants of plant health and productivity has been also known since long time^4,14^. Less is known about the plant-species specific component on the structure and function of raw-eaten plants and their relationship to health^15,16^.

One example for which a plant-family specific structure and function of the associated microbiome is already studied are *Brassicaceae* ^17^. All family members are characterized by glucosinolates (GLSs) that are part of the effective defense mechanisms of the plant^18^. Moreover, they are known for a bacteria-dominated composition of the microbiome and harbor no mycorrhiza^19,20^. The hydrolysis of GLSs into highly active breakdown products, mostly isothiocyanates (ITC) and nitriles, is caused by myrosinase activity^21^. Those volatile breakdown products are utilized in biofumigation processes, where *Brassica* residues are incorporated into soil as they provide suppressive or control effect against nematodes and soil-borne fungal pathogens like *Verticillium longisporum* ^22,23^. Interestingly, GLSs are also involved in human health issues. Indeed, the GLS metabolism has become increasingly important over the past decade due to the exploration of anti-cancer activity of ITCs^24^. However, it was demonstrated that plant myrosinase is inactivated by long-term storage or cooking^25^. Since humans consume their vegetables often cooked, the GLS-metabolizing ability of bacteria^26^ has recently aroused scientific interest. Some authors consider the addition of myrosinase-active bacteria to a *Brassica* rich diet to supplement inactivated plant myrosinases^27^. While the majority of bacterial strains known to exhibit myrosinase activity are ubiquitous inhabitants of the human intestinal tract^27^, still little is known about myrosinase-active bacteria colonizing edible plants tissues. We hypothesized that due to their GLS content *Brassica* vegetables harbor a very specific microbiota containing also myrosinase-active bacteria.

The present study unravels diversity and compositions of the microbiomes of usually eaten parts of seven different *Brassica* vegetables, focusing on two specific, health promoting functions of the microorganisms: i) myrosinase-activity, providing potential cancer-preventing properties for humans and ii) antagonistic activity towards microbial pathogens on crop plants. For the latter, we selected *Verticillium longisporum* as model pathogen, which is an emerging, high-risk pathogen of *Brassicaceae* ^28^ and its outbreaks are strongly correlated to the GLS content of the plant^23^. Vegetable samples were taken from the usually eaten plant parts. We considered whereas the food processing, associated with the point of sale, influences the microbial community composition, and consequently affects human health, by comparing vegetables purchased at a local farmers market and a supermarket.

## Results

### The structure of the microbiomes associated with *Brassica* vegetables

The overall bacterial community, assessed by 16 S rRNA gene amplicon sequencing, contained a total of 2,880,421 reads (forward and reverse reads). After removing chimeras, mitochondrial, chloroplast and unassigned sequences, 896,833 sequences remained, resulting in a total of 10,458 OTUs. The normalized dataset contained 42 bacterial phyla, resulting in 257 bacterial orders. The highly abundant fraction of the microbiome of all vegetables (>1% abundance) was dominated by *Proteobacteria* (75%), followed by *Bacteriodetes* (19%) and *Actinobacteria*, *Verrucomicrobia* and *Firmicutes* each with 2%.

### The influence of plant genotype and processing pathway on the vegetable microbiomes

**Figure 1:**
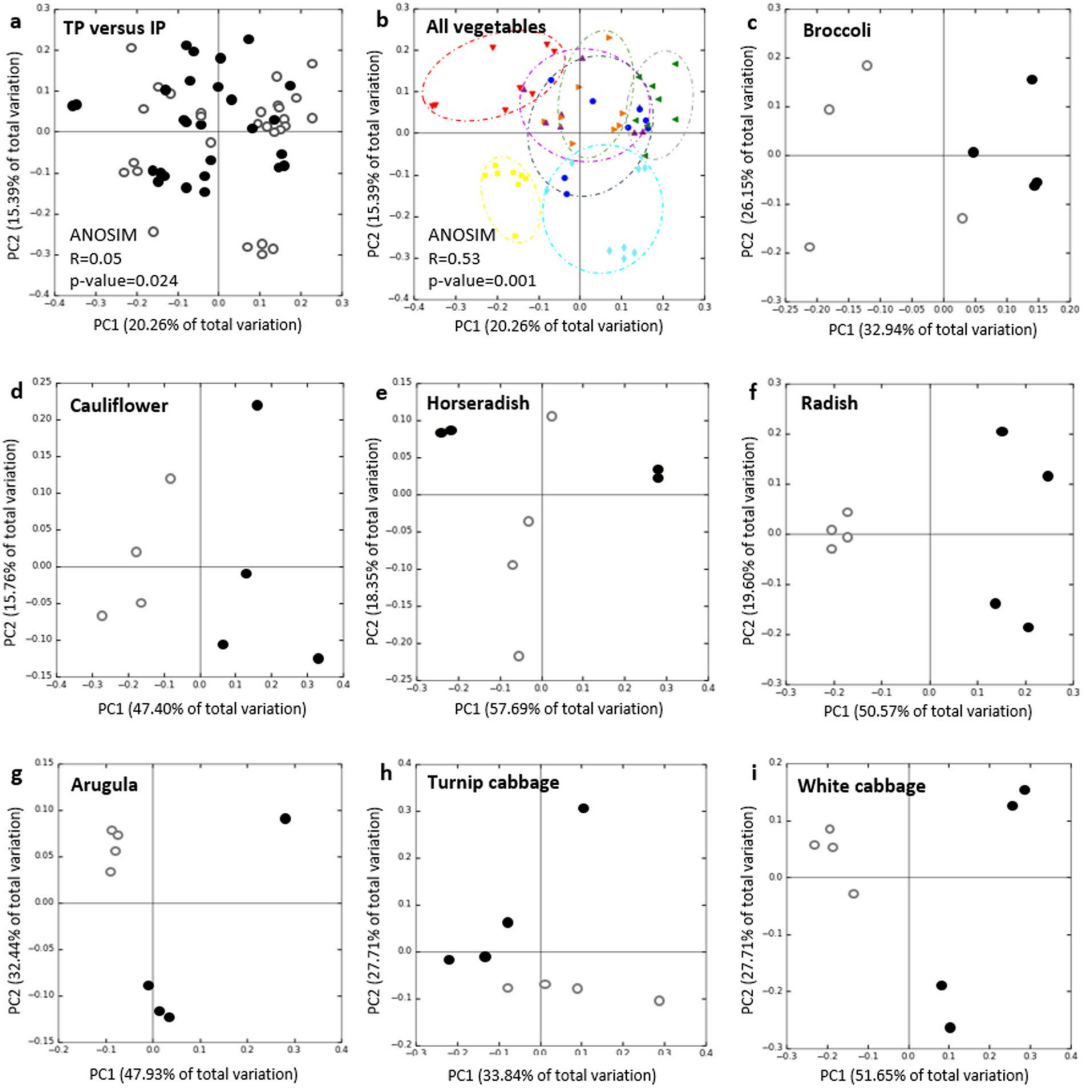
Beta-diversity metrics of bacterial 16S rRNA gene of the vegetable samples visualized by PCoA plots. Community clustering is based on Bray-Curtis dissimilarities (weighted UniFrac) in the relative abundance of OTUs. (**a**) Bacterial community composition of the pooled traditionally processed samples (TP; filled circles) and the pooled industrially processed samples (IP; open circles). (**b**) Microbiome variations derived from the vegetable genotype. The colors of the symbols denote for the different vegetables: horseradish (red), arugula (yellow), radish (turquoise), white cabbage (blue), turnip cabbage (green), cauliflower (orange) and broccoli (purple). Circles were drawn for a better visualization of the clusters. ANOSIM describes impact and significance of processing pathway (R = 0.05; p-value = 0.024) and plant genotype (R = 0.53; p-value = 0.001) on the microbiome structure. (**c**–**i**) Partitioning of bacterial communities associated with the processing equivalents of each vegetable type. Filled circles denote for traditionally processed samples, open circles show industrially processed samples.

Between-sample differences of the microbiota within traditionally (TP) and industrially (IP) processed vegetables, purchased at a local farmers market and a supermarket, respectively, were analyzed to study relationships among the vegetable samples based on phylogenetic composition. When the samples were pooled by their processing pathway, no clusters were observed in the PCoA plots (Fig. 1a). In contrast, when the microbiomes of the vegetables cultivars were pooled by their genotype, separated clusters for several genotypes were observed (Fig. 1b). ANOSIM (Analysis of similarities) suggests that the plant genotype (R = 0.53; p = 0.001) has a higher and more significant influence on the microbiome structure than the processing pathway (R = 0.05; p = 0.024). Clearest separation according to the principle coordinates was observed for the microbiomes of horseradish, radish, turnip cabbage and arugula. Microbiomes derived from white cabbage, cauliflower and broccoli clustered close together along all three coordinates, separated from the remaining vegetables.

A strong cultivar-specific impact of food processing on the microbiome was observed when the equivalents of each vegetable type were compared (Fig. 1c–i). The percentages of shared OTUs ranged from 56.2% for white cabbage to 64.7% for horseradish equivalents (Supplementary Table S1). In order to investigate what taxa are influenced by food processing the most, Kruskal-Wallis test on the most abundant taxa (abundancy over 3% on genus level) was performed. Twenty-six taxa were found to be significantly higher represented in either traditionally or industrially processed vegetables (Table 1). Due to their high abundance, especially *Acinetobacter* and members of *Oxalobacteriaceae* are suggested to be typical for industrially processed vegetables, while *Sphingopyxis, Sphingomonas, Pseudomonas* and *Pseudoxanthomonas* are indicators for traditionally processed vegetables. However, these indicator taxa were also strongly genotype-specific. Highest number of taxa with significantly higher abundance in one of the two sample equivalents was found for radish and arugula, while no taxa meeting these criteria were found for horseradish samples.

**Table 1:** Significant differences in relative abundances between traditionally and industrially processed equivalents of each vegetable type.

Vegetable	Taxonomy*		Relative abundance (%)**	
	Family	Genus	Traditionally processed	Industrially processed
Radish	*Sphingomonadaceae*	*Sphingopyxis*	**4.3**	0.5
	*Sphingomonadaceae*	*Sphingomonas*	**3.5**	0.8
	*Xanthomonadaceae*	*Pseudoxanthomonas*	**3.0**	0.2
	*Oxalobacteraceae*	unclassified	1.2	**12.8**
	*Sphingobacteriaceae*	*Pedobacter*	1.0	**10.6**
	*Flavobacteriaceae*	*Flavobacterium*	2.8	**7.5**
	*Micrococcaceae*	unclassified	1.5	**5.8**
	*Comamonadaceae*	*Methylibium*	0.1	**3.8**
Cauliflower	*Pseudomonadaceae*	*Pseudomonas*	**21.8**	9.0
	*Enterobacteriaceae*	unclassified	**10.9**	2.1
	*Moraxellaceae*	*Acinetobacter*	2.4	**20.3**
	*Sphingomonadaceae*	*Sphingomonas*	2.9	**18.5**
Turnip cabbage	*Bacillaceae*	*Bacillus*	0.4	**6.9**
	*Leuconostocaceae*	*Leuconostoc*	0.1	**6.0**
Arugula	*Oxalobacteraceae*	unclassified	**4.6**	1.7
	*Xanthomonadaceae*	unclassified	**3.8**	1.1
	*Moraxellaceae*	*Acinetobacter*	3.8	**11.9**
	*Oxalobacteraceae*	*Janthinobacterium*	1.6	**4.3**
	*Exiguobacteraceae*	*Exiguobacterium*	1.2	**3.3**
	*Sphingobacteriaceae*	*Sphingobacterium*	0.5	**3.3**
	*Shewanellaceae*	*Shewanella*	0.6	**2.6**
White cabbage	*Enterobacteriaceae*	unclassified	**14.7**	4.5
	*Alcaligenaceae*	*Achromobacter*	**5.3**	0.1
	*Methylobacteriaceae*	*Methylobacterium*	0.6	**8.1**
	*Cytophagaceae*	*Runella*	1.4	**3.0**
Broccoli	*Oxalobacteraceae*	*Janthinobacterium*	**5.6**	1.3

*Only taxa with abundance greater than 3% in one group, showing significant difference in abundance (p < 0.05) between the two groups are shown. No taxa meeting those criteria were found for horseradish. **The values indicating higher abundances of the taxa at the respective processing pathway are highlighted in *bold*.

**Figure 2:**
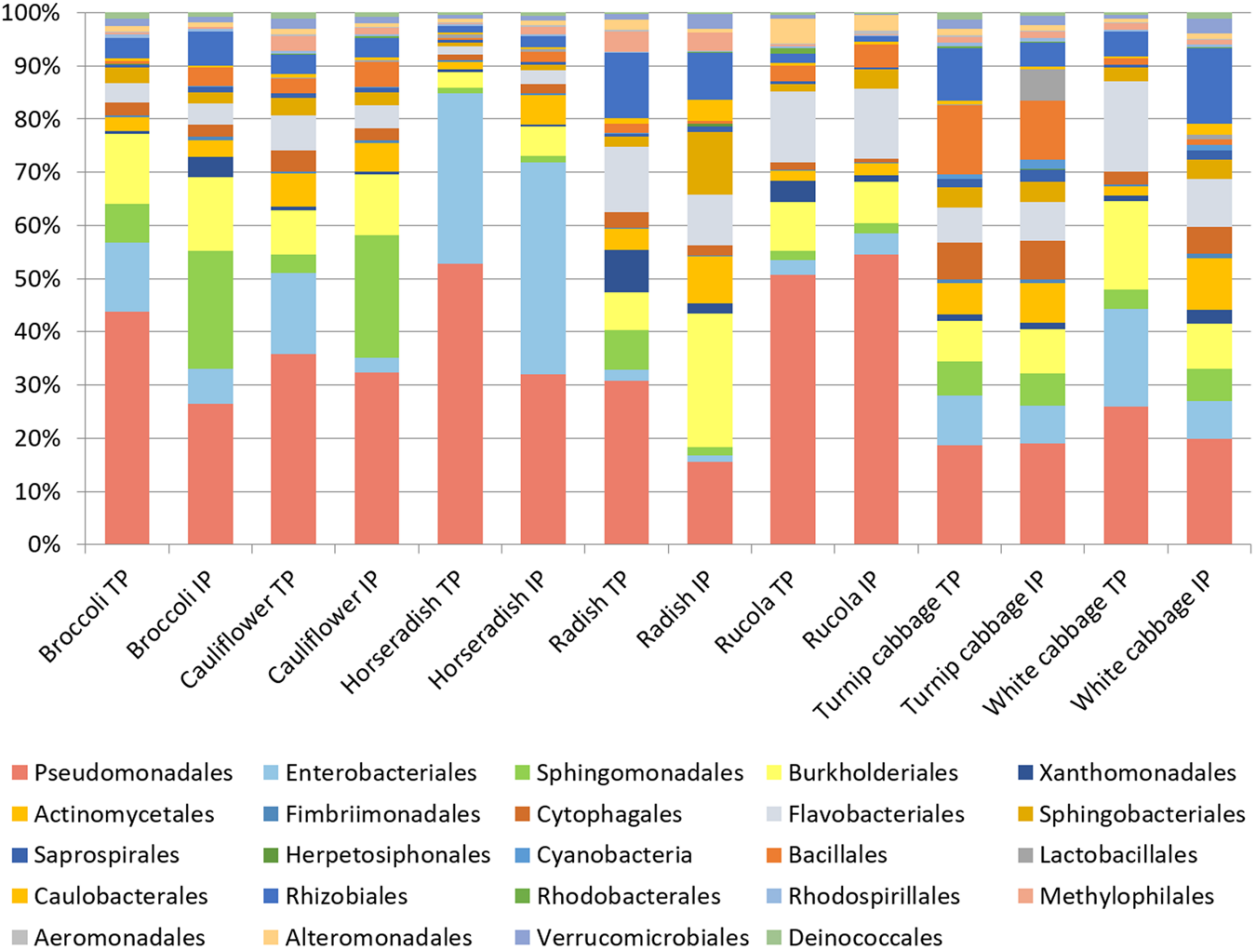
Taxonomic classification of the microbiota inhabiting the processing equivalents of the vegetables types. Bar charts represent the bacterial composition on order level by using a threshold of at least 1% abundance. Abbreviation: TP, traditionally processed; IP, industrially processed.

Analyzing the within-sample variations of all vegetable microbiomes showed no significant differences for the 24 most abundant orders (>1% abundance; Fig. 2); however, the percentage distribution of those orders varied between the different vegetable genotypes. Bacterial orders from horseradish were found to be most unevenly distributed among all vegetable samples with *Pseudomonadales* and *Enterobacteriales* constituting more than 72% of the total bacterial community. Turnip cabbage indicated the most even distribution among the investigated samples, as 80% of the total community was represented by 13 different bacterial orders. Percentage distribution of the most abundant orders was highly similar for processing equivalents of each vegetable type.

Alpha-diversity metrices were used to observe differences in OTU richness among the 14 vegetable samples (Supplementary Figure S1a). The OTU richness of the samples in the normalized dataset ranged from 180 OTUs for industrially processed arugula to 325 OTUs for traditionally processed cauliflower; the latter contained significantly higher bacterial diversity than its industrially processed equivalent (Supplementary Table S2). Comparing the OTU richness of the samples pooled by their purchase origin revealed no significant differences (Supplementary Figure S1b).

**Figure 3:**
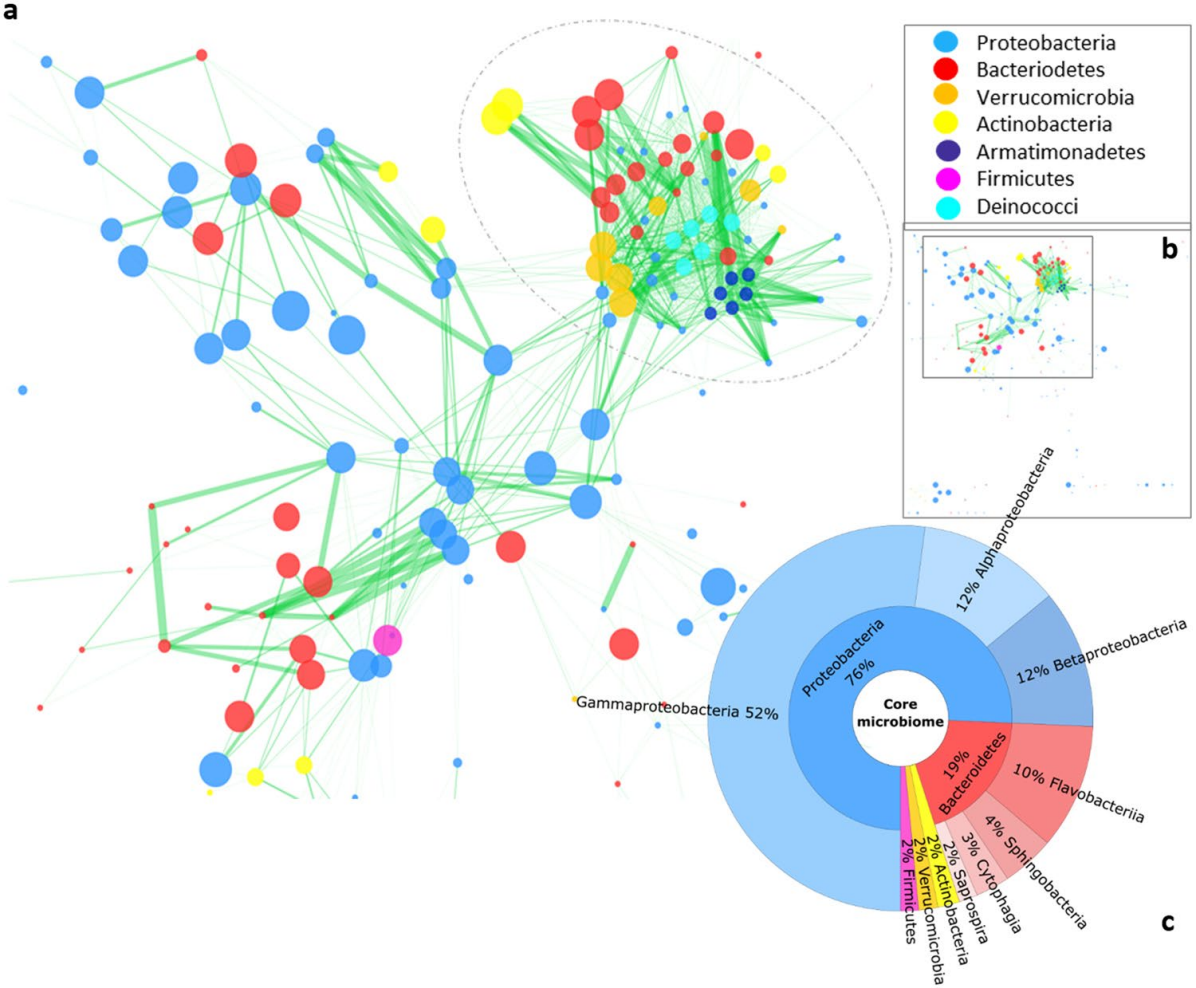
Significant co-occurrence relationships among the *Brassica* vegetable microbiomes. Only significant interactions are shown (p < 0.0004; q < 0.0004). Nodes correspond to OTUs and the size of the nodes corresponds to the relative abundance of the OTUs in the dataset. Green edges represent significant positive Spearman’s correlation between the pairs of OTUs. The edge width indicates the significance of interaction. The color of the nodes indicates the corresponding phylum of the OTUs as described in the legend on the right. Circle was drawn to highlight the denser part of the network. The Figure in (**b**) shows the full network highlighting the part of the network zoomed in (**a**). (**c**) Core microbiome shared by all *Brassica* samples representing the taxonomic classification up to class level in ascending order using a threshold of 1% abundance.

In order to elucidate co-occurrence interactions within the microbial community of *Brassica* vegetables, a spearman co-occurrence network was constructed (Fig. 3a). The network was examined for statistically significant (p < 0.0004; q < 0.0004) co-occurrence and co-exclusion patterns among all OTUs; exclusively positive interactions were found. Some OTUs with loose interactions that do not fit into the condensed structure were observed as well (Fig. 3b). The network contains one very dense part (designated by a circle around the network part in Fig. 3a), indicating intense interconnection of involved taxa, and a more dispersed part. The dense part is, except for *Bacteriodetes*, almost exclusively represented by OTUs assigned to taxa with low abundance in the vegetable core microbiome (Fig. 3c): *Verrucomicrobia, Actinobacteria, Armatimonadetes* and *Deinococci*. The more dispersed part mainly consists of *Proteobacteria* and *Bacteriodetes* OTUs, which occur in high abundances in the core microbiome.

### Plant genotype- and processing pathway-specific indicator taxa

The 16S rRNA gene library of the vegetable samples was investigated for strains commonly known for their beneficial impact on human health. Abundance of *Sphingomona*s was significantly higher in radish TP, but lower in cauliflower TP in comparison to their IP equivalent. Lactic acid bacterium *Leuconostoc* occurred significantly more often in turnip cabbage IP (Table 1). Abundance of other known beneficial taxa like *Lactobacillus* or *Bifidobacterium* was not significantly different between the vegetables (results not shown); the same applies for *Bacteroidetes*, which were highly abundant in all vegetables. Genera containing opportunistic or potential human pathogens (*Clostridium, Stenotrophomonas, Legionella, Neisseria, Rickettsia, Staphylococcus* and *Streptococcus*) were found as well; however, in extremely low abundance and with no significant difference between the processing equivalents (results not shown). A variety of taxa, like *Pseudomonas, Klebsiella, Serratia, Agrobacterium, Azospirillum, Burkholderia* and representatives of *Rhizobiaceae*, described for their plant beneficial effects were found in the vegetable samples; differences between the processing equivalents were not significant (results not shown).

### The microbiome of *Brassica* vegetables as a source for myrosinase activity

The myrosinase-active fraction of the microbiome, suggested to afford chemo-protection for humans, was investigated to evince potential health promoting functions of the vegetable consumption. In total one myrosinase-active strain, *E. cloacae* KS50 (NCBI accession: KY784664), was isolated from the bacterial communities of the 14 vegetable samples. Since cultivation of myrosinase-active bacteria on sinigrin-containing M9 minimal salts agar plates was inefficient, screening started with the incubation of the whole bacterial community of each vegetable sample in M9 minimal salts liquid media, with sinigrin as a sole carbon source. The bacterial collection of turnip cabbage IP revealed the single positive result in the GOD-POD coupled enzyme assay (myrosinase activity: 33.25 U ml^−1^). The suspension was spread on selective agar plates; one single strain (KS50), with high similarities (up to 100%) to several members of the *E. cloacae* complex, formed colonies on the plates. In the second step, myrosinase activity of cell-free *E. cloacae* KS50 was demonstrated in triplicate: i) myrosinase activity of 2.343 U ml^−1^ in GOD-POD coupled enzyme assay, ii) growth on selective agar plates and iii) myrosinase activity of 16.583 U ml^−1^ calculated by degraded sinigrin using HPLC. The putative myrosinase gene of *E. cloacae* KS50 was amplified using a PCR with degenerate primers. The translated amino acid sequence of the resulting myrosinase fragment revealed 100% sequence similarity to the 6-phospho-β-glucosidase *bglA* (NCBI accession: AEW75128.1) of *E. cloacae* EcWSU1. Searching for the sequence of *E. cloacae* KS50 in the amplicon database of all vegetable samples revealed eight hits. A range of microbiota is able to process GLSs, including particularly *Enterobacteriaceae* ^26^, but also *Lactobacillaceae* ^29^, *Bacillaceae* ^30^ and *Bifidobacteriaceae* ^31^. GLS-degrading enzymes are, however, presumably strain specific and active inducible^27^. Therefore, assigning this specific function to the present amplicon library of the vegetable microbiomes, based on 16 S rRNA gene sequencing, was found to be rather not sensible.

Genomic libraries of phyllosphere, rhizosphere and soil metagenome of *E. sativa* plants, as well as rhizosphere and bulk soil of four *B. napus* plants, were screened for the bacterial myrosinase genes *bglA* and *ascB* (NCBI accession: EGT69672.1) (Supplementary Figure S2). *E. sativa* phyllosphere showed 152 and 92 hits, rhizosphere 55 and 58 hits and bulk soil 24 and 14 hits, for *bglA* and *ascB*, respectively. Mean value of hits in *B. napus* rhizosphere was 19.5 ± 5.8 and 17.5 ± 7.3 as well as 1 ± 0 and 1 ± 1.4 hits in corresponding bulk soils. Assigning the sequences of recovered hits to their taxonomic affiliation by comparison to NCBI nucleotide database resulted in sequence similarities to several members of *Enterobacteriaceae* and some *Firmicutes*, frequently described for possessing myrosinase activity^27^. BLASTx alignment of all hits to NCBI nonredundant protein database showed 100% sequence similarity to 6-phospho-β-glucosidases (bacterial myrosinase).

### The microbiome of *Brassica* vegetables as a source for biological control agents (BCA)

Beneficial effects for *Brassica* plants were investigated by screening vegetable-associated microbiota for antagonistic potential towards *V. longisporum* and *in vivo*-trials using *B. napus* as model host organism. In total 560 bacterial strains were tested; five of them, isolated from traditionally processed vegetables, were identified to combat *V. longisporum* in a dual-culture plate assay: *Pseudomonas fluorescens* F2 (NCBI accession: KY784660)*, Serratia plymuthica* F20 (NCBI accession: KY784659) and *Pseudomonas prote gens* F37 (NCBI accession: KY784662) isolated from cauliflower, *Pseudomonas azotoformans* RU40 (NCBI accession: KY784663) isolated from arugula and *Pseudomonas fragi* W31 (NCBI accession: KY784661) isolated from white cabbage (Table 2). The mean zones of inhibition from the five strains were not significantly different from each other (p < 0.05) as identified by one-way ANOVA Post-hoc Tukey HSD test. The ability of the antagonistic strains to promote plant health was investigated by evaluating the germination rate and comparing the weights of the green parts and the roots of the seedlings with the untreated control plants after growth under gnotobiotic conditions for 14 days. It was attempted to keep the cell concentration constant by adjusting an OD_600_ value of 10 for each strain. Nevertheless, the concentration of the live cells, measured by calculating the CFU ml^−1^, varied between strains. Interestingly, *S. plymuthica* F20 showed the lowest live cell count in the suspension used for the bio-priming, but indicated the second highest level of cell abundances in the roots. All strains had neutral to mildly positive effect on the growth of the oilseed rape seedlings, while only bio-priming with *P. fluorescens* F2 resulted in a significant promotion of plant growth (Table 2). No significant effect on the germination rate was determined.

**Table 2:** Impact of selected strains on the *B. napus* seedlings grown in germination pouches.

	*V*. *lonisporum*: zones of inhibition (mm)^†^	Priming concentration (log_10_ CFU ml^−1^)	Abundances on the root (log_10_ CFU [g] root^−1^)	Weight of the roots (10 plants^−1^ [mg])	Weight of green parts (10 plants^−1^ [mg])	Germination (%)
Control		0.0	0.0 ± 0.0	256 ± 97	337 ± 72	75 ± 2.9
*P. fluorescens* F2	2.8 ± 1.3^a^	8.9	9.5 ± 0.1	758 ± 264*	758 ± 187*	73 ± 1.5
*P. protegens* F37	2.8 ± 3.0^a^	8.4	11,6 ± 0.1	263 ± 80	370 ± 26	66 ± 1.4
*S. plymuthica* F20	7.6 ± 0.4^bc^	7.6	10,6 ± 0.2	276 ± 109	509 ± 86	86 ± 2.2
*P. fragi* W31	4.0 ± 2.9^ab^	9.6	10,0 ± 0.6	272 ± 49	434 ± 70	89 ± 1.1
*P. azotoformans* RU40	9.8 ± 1.3^c^	10	10,4 ± 0.1	219 ± 67	393 ± 72	88 ± 1.1

^†^The common letters following the mean zones of inhibition of the five independent replicates indicate non-significant difference for each strain (estimated with ANOVA Post-hoc test Tukey HSD at p = 0.05). *Asterisk* after the values denote for significant difference to the non-primed control plants according to the *t*-test for independent samples (p < 0.05).

**Figure 4:**
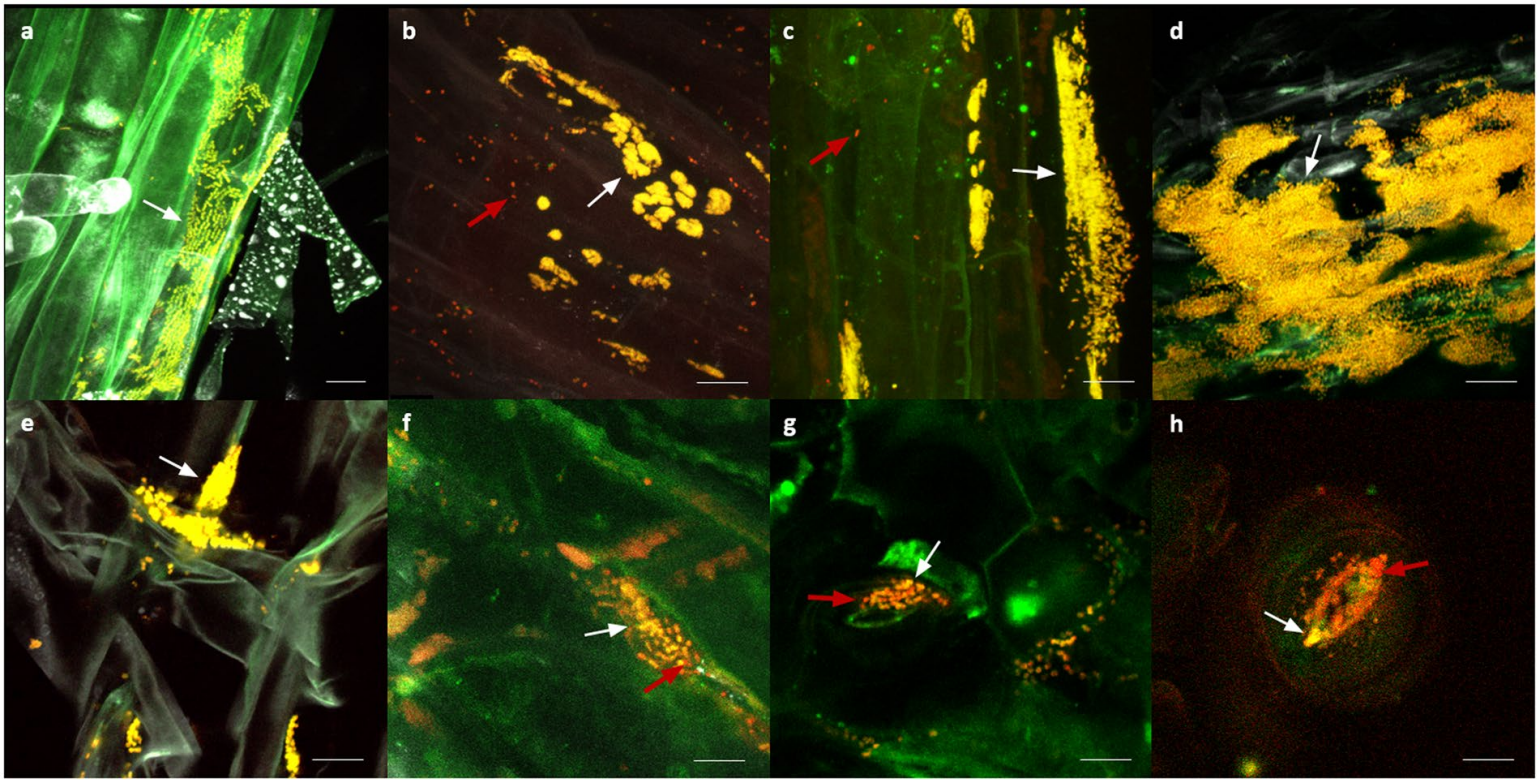
FISH/CLSM visualization of antagonistic bacterial strains colonizing oilseed rape seedlings. Expected colonies, denoted by white arrows, appear yellow due to overlay of the fluorochromes Cy3 and Cy5. Red arrows indicate other bacterial organisms which are presumably seed-borne. (**a**) Rhizosphere with *P. fluorescens* F2; (**b**) Rhizosphere with *S. plymuthica* F20; (**c**) rhizosphere with *P. protegens* F37; (**d**) rhizosphere with *P. fragi* W31; (**e**) rhizosphere with *P. azotoformans* RU40; (**f**) phyllosphere with *P. fluorescens* F2; (**g**) phyllosphere with *P. fragi* W31; (**h**) phyllosphere with *P. azotoformans* RU40. The bar represents 10 µm.

Colonization patterns of the antagonistic strains in oilseed rape seedlings were visualized using FISH/CLSM (Fig. 4). High abundances of all strains were observed especially in the middle and upper parts of the roots. All tested strains formed large micro-colonies in the root tissue (Fig. 4a–e). Only a few phyllospherically located colonies appeared in the tissues of the seedlings bio-primed with *P. fluorescens* F2 (Fig. 4f), *P. fragi* W31 (Fig. 4g) and *P. azotoformans* RU40 (Fig. 4h) occurring inside and around stomata. Some contaminations were observed in the plant tissue, indicated by red fluorescent spots, which are assumed to be seed-borne strains; their majority was observed in the seedlings’ phyllosphere.

### Bacteria antagonistic towards *Verticillium* within the *Brassica* microbiome

**Figure 5:**
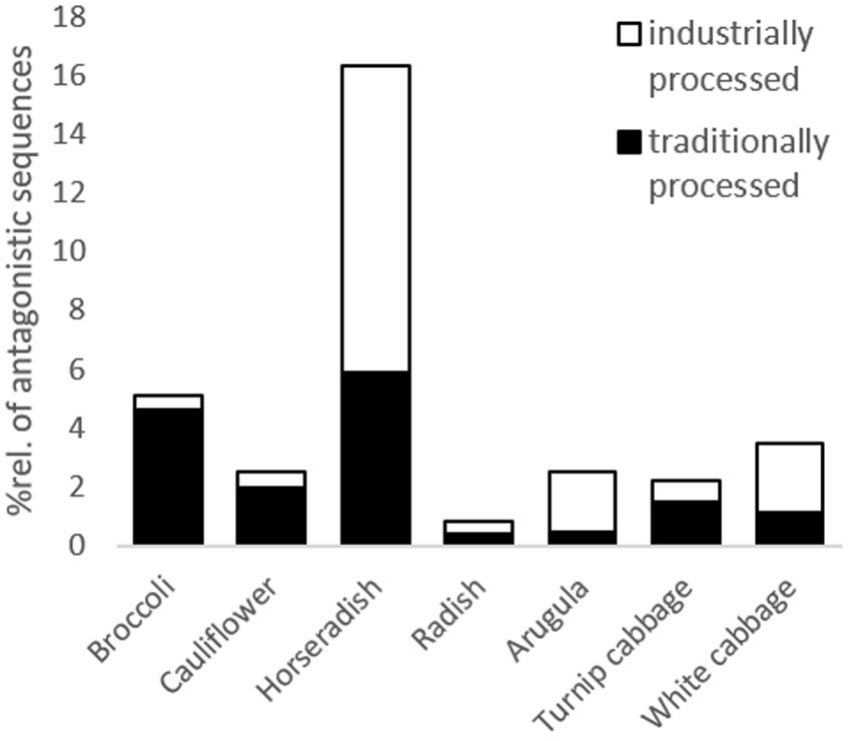
*Verticillium*-antagonistic sequences within the vegetable microbiomes. Columns represent the relative amount of sequences (%) assigned to isolated *Verticillium*-antagonistic bacteria (listed in Table 2) in the 16S rRNA gene library of each vegetable genotype. Columns are subdivided in traditionally processed (filled columns) and industrially processed (open columns) samples.

In order to determine the biocontrol potential of the *Brassica* microbiome, the 16S rRNA gene amplicon library was screened for the isolated strains with inhibitory effect towards *V. longisporum* (Table 2). In total, 4.1% of the vegetable microbiota were represented by *Verticillium*-antagonistic isolates. Among them, *S. plymuthica* F20 was most frequent (96.74%), followed by *P. fragi* W31 (3.09%), *P. azotoformans* RU40 (0.13%), *P. protegens* F37 (0.03%) and *P. fluorescens* F2 (0.01%). Comparing vegetable genotypes, horseradish showed the highest antagonistic potential (16.3% of sequences were assigned to *Verticillium*-antagonistic isolates) followed by cauliflower (12.9%), broccoli (5.1%), white cabbage (3.5%), arugula (2.5%), turnip cabbage (2.2%) and radish (0.8%). Traditionally processed broccoli, cauliflower and turnip cabbage contained more *Verticillium*-antagonistic sequences than their industrially processed equivalent; the opposite was true for horseradish, radish, arugula and white cabbage (Fig. 5).

### Potential function of the microbiomes associated with *Brassica* vegetables

In order to investigate vegetable-associated microbiota for health-affecting functions, PICRUSt analysis was performed (Supplementary Figure S3). No differences for the functions correlated to human diseases were observed comparing the pooled traditionally to the pooled industrially processed samples. The functional differences associated with the processing pathways were restricted to the vegetable type; the KEGG pathway for ‘human diseases’ was found to be higher in broccoli TP and white cabbage IP in comparison to their processing equivalents, especially for the KEGG subcategories ‘cancers’, ‘cardiovascular diseases’, ‘immune system diseases’, ‘infectious diseases’, ‘metabolic diseases’ and ‘neurodegenerative diseases’.

## Discussion

**Figure 6:**
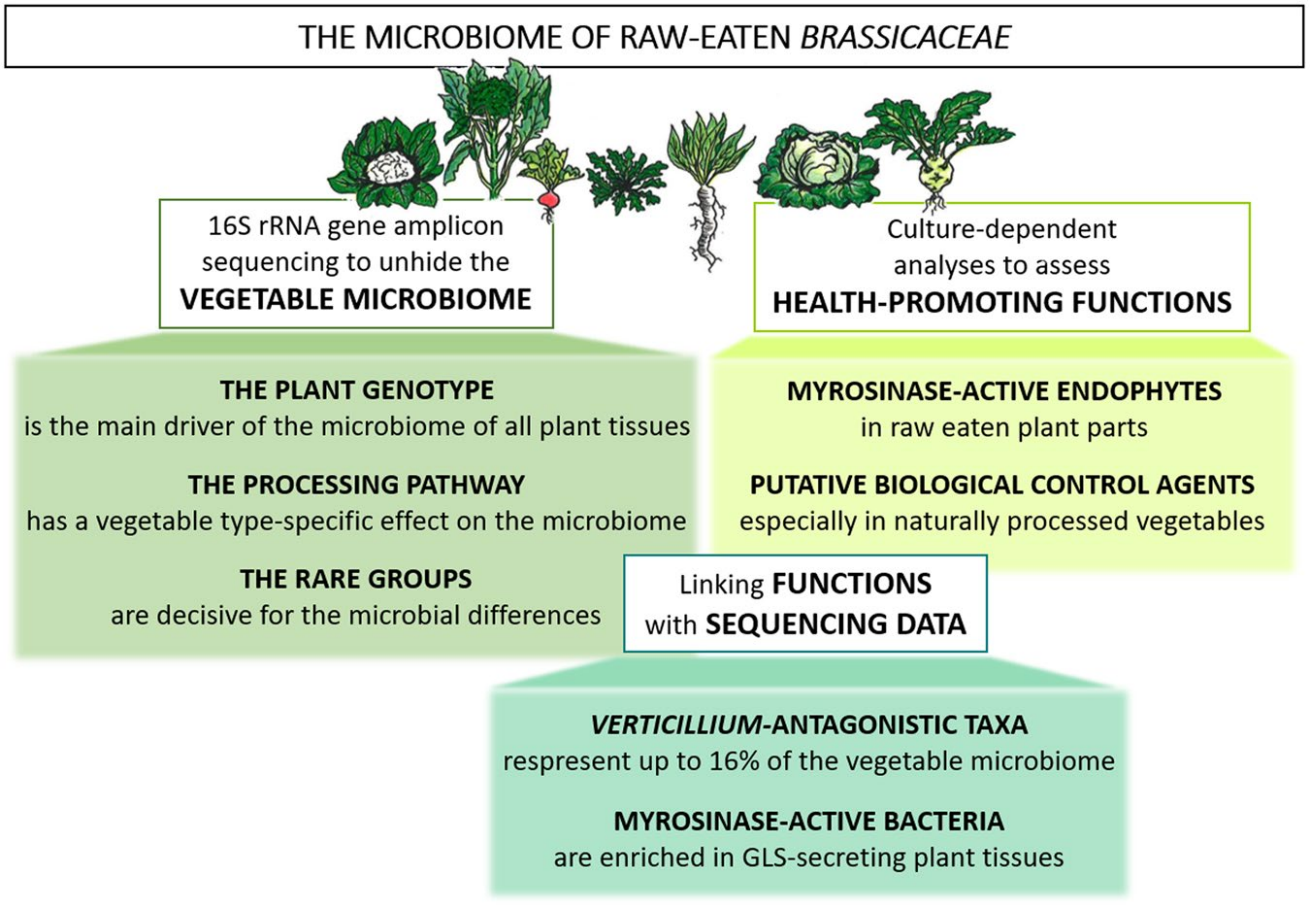
Overview of the present study investigating the microbiome of raw-eaten *Brassicaceae*. The composition and the crucial drivers of the vegetable microbiomes were determined using cultivation-independent 16S rRNA gene amplicon sequencing. Cultivation-dependent approach was performed to isolate bacteria from vegetables that execute specific health-promoting functions: myrosinase activity proving potential anti-cancer effects in humans and antagonistic activity towards *V. longisporum*. Additionally, in order to locate bacterial myrosinase genes in different parts of *Brassica* plants, the metagenomes of *E. sativa´s* and *B. napus´* phyllosphere, rhizosphere and bulk soil were investigated.

By investigating usually eaten parts of each vegetable type, combining phyllosphere, endosphere and rhizosphere habitats, a comprehensive microbial diversity was revealed. With that, we demonstrated that the plant genotype is the main driver of the microbiome of all plant parts. The processing pathway was found to play a subordinate role for the microbiome composition. Furthermore, we suggest *Brassica* vegetables to be a host for a great diversity of health-promoting bacteria. The observations gained from the multiphasic approach of this study are illustrated in Fig. 6.

All vegetable microbiomes share the same high abundant taxa, while differences were found within the rare groups. Van Elsas *et al*. (2012) and Cardinale *et al*. (2015) suggest that a network of rare groups can enhance a barrier effect against plant pathogens^32,33^. The co-occurrence network patterns of *Brassica* vegetables, showing intense structuring of low abundant taxa, confirm the importance of those rare groups. The microbiomes of all unpeeled and phyllosphere samples (white cabbage, cauliflower and broccoli) resembled each other and differed from rhizosphere samples, while the microbiome of arugula stands alone. These differences might be due to high contents of phytochemicals in arugula^34^ and also the plants´ morphology, revealing a more exposed habitat for microbiota than investigated parts of other phyllosphere samples. The impact of GLSs on plant microbiomes has previously been described^35,36^. We determined the coherence of GLS and microbiota in arugula by comparing metagenomics samples of phyllosphere, rhizosphere and bulk soil. The abundance of bacterial myrosinase genes was highly enhanced in the phyllosphere in comparison to the corresponding bulk soil, suggesting supplementary advantage of *Brassica* plants in biofumigation processes. We assume myrosinase-active bacteria to adapt to their environment by active induction of the enzyme^27^, leading to specialization in a GLS-rich habitat. Whether this specialization creates only advantages for the microorganism in nutrient supply or if the bacterial accumulation is plant-triggered as well, remains uncertain.

Bacterial myrosinase activity as a specific human beneficial function was demonstrated by isolating myrosinase-active *E. cloacae* KS50 from the endosphere of turnip cabbage. Hitherto, bacterial myrosinase activity was ascribed to members of the human gut; the present study was the first to isolate a strain directly from the usually eaten parts of a vegetable. Cultivating myrosinase-active bacteria proved to be difficult which might be due to the observation that the GLS uptake operates less efficient than the uptake of other sugars^27^. Despite that, we were surprised to isolate only one myrosinase-active bacterium out of the highly diverse vegetable microbiomes. The comparison of two recent studies^37,38^, reporting different genetic origins of bacterial *β*-glucosidases with myrosinase activity, suggests higher diversity of bacterial myrosinase genes with different substrate specificity. Furthermore, we assume that myrosinase activity might be dependent on a co-metabolic pathway of associated bacteria since the microbial community of turnip cabbage revealed 10-fold higher values for myrosinase activity than the pure culture of *E. cloacae* KS50. Composition and metabolism of the human gut microbiome is shaped by the diet^39^ and myrosinase activity was previously determined for intestinal *E. cloacae* ^26^. Assuming that the GLS-degrading ability of *E. cloacae* (and other intestinal bacteria) has originated in cruciferous plants, we suggest *E. cloacae* to be a representative of a long live relationship between edible plants and the gut microbiome, since the bacterium fulfills the same function in both hosts, namely the degradation of GLSs for nutrient supply; this leads to enhanced defense mechanisms for the holobiont. Some authors promote the idea of adding myrosinase-active bacteria to a *Brassica*-rich diet to increase the anti-cancer effects of the GLS hydrolysis^27^. This however, is a subject of further investigations and clinical studies due to the lack of knowledge on bacterial myrosinase activity, the variety of GLS formation in plants and the complex interplay of mechanisms occurring in the human gut.

A general tendency of human health supporting bacteria occurring more frequent in traditionally or industrially processed vegetables was not observed; neither on a taxonomical, nor on a functional level. Differences were restricted to rare groups and were only observed between the processing equivalents of each vegetable type. Specific health affecting property is assigned to the overall high abundance of *Gammaproteobacteria* and *Bacteriodetes* in all *Brassica* vegetables. The former show high variability in their functions, the latter are frequently described for their beneficial impact in human gut. Other health promoting taxa like *Sphingomonas, Leuconostoc, Lactobacillus* and *Bifidobacterium* occurred in all samples investigated. Commonly described human pathogens were found as well; however, their abundance was exceedingly low compared to the beneficial taxa mentioned above. An adverse health effect for humans is therefore not expected; moreover, it is assumed that a highly diverse microbiome on fresh produce, containing a moderate amount of opportunistic bacteria as well, might serve as a stimulus for the human immune system, also described as ‘natural vaccination’^16^.

The effects of certain bacteria can be ambivalent in plants and humans. High abundance of plant-beneficial strains could be a substantial disadvantage for human health; hence, BCAs have to be selected with caution. For that reason, we chose raw-eaten vegetable parts as isolation source for putative BCAs. All of the five strains combating *V. longisporum* were isolated from traditionally processed vegetables. Plants themselves influence the composition of their microbial community to some extent^40^. Regarding to this, we assume that due to the application of chemical fertilizers and pesticides, plant beneficial bacteria are less in demand for industrially processed vegetables. Especially horseradish bears high biocontrol potential; one sixth of all sequences within the horseradish microbiome is represented by *Verticillium*-antagonistic taxa. Furthermore, gluconasturtiin and sinigrin, the main GLSs of horseradish, are the precursors of ITCs described for powerful inhibitory effects towards various plant-pathogenic fungi^41^. At this point it has to be mentioned that the vegetable-associated microbiome was merely screened for the sequences of the five *Verticillium*-antagonistic bacteria isolated in the present study; the biocontrol potential is therefore suggested to be far higher. Special tasks in the vegetable microbiome might be attributed to isolated *S. plymuthica* F20, since the amount of sequences in the amplicon library was particularly high (<42,000 hits). Strains of *S. plymuthica* are well-characterized antagonists and BCAs from the oilseed rape microbiome^42,43^. *P. fluorescens* F2 fulfilled three *in vitro* requirements of a putative BCA; the biocontrol potential has to be confirmed under natural conditions. Colonization capacity of the strains in the roots were confirmed by FISH/CLSM while only few colonies were observed in the phyllospheric parts of the seedlings which might be due to the fact that two weeks of incubation are not enough time for the strains to colonize all plant parts. The phyllosphere was, however, colonized by endophytic seed-borne strains. Seed-borne endophytes establish intimate relationships with their host plants^44^; we speculate that those bacteria are better adjusted to the host plant, allowing a faster colonization of the phyllospheric parts in comparison to the strains used for bio-priming.

## Conclusion

Distinctive knowledge of the microbiota inhabiting raw-eaten vegetables and fruits is of crucial importance; not only for health and productivity of crop plants but also for humans as consumers. Our results indicate the plant genotype to be the main driver of the microbiome composition of all plant parts and plant-associated bacteria to improve the health benefits of the vegetable consumption. Glucosinolates, the unique secondary metabolites in *Brassicaceae*, seem to play a crucial role, which has to be considered in further breeding activities. Furthermore, we promote the idea of plants´ targeted enrichment of microbiota and the importance of *Brassica* holobionts in biofumigation processes, by detecting bacteria with active myrosinase and antagonistic potential towards *V. longisporum*.

## Methods

### Sampling and experimental design

The following cruciferous vegetables, each one in four biological replicates, were purchased at a local farmers market (TP) and a supermarket (IP), respectively: *Brassica oleracea* f. *alba* DC. (white cabbage), *B. oleracea* var*. italica* Plenck (broccoli), *B. oleracea* var. *botrytis* L. (cauliflower), *Eruca sativa* Mill. (arugula), *Raphanus sativus* L. (radish), *B. oleracea* var*. gongylodes* L. (turnip cabbage) and *Armoracia rusticana* G.Gaertn., B.Mey., & Scherb. (horseradish). Fresh vegetables were purchased on 24^th^ and 25^th^ of September 2015 in order avoid previous long-term storage. All vegetables were washed with deionized water and the commonly eaten parts of each type were used, giving a broad diversity of microhabitats: the inflorescent parts of broccoli and cauliflower; the leafs of arugula, the roots of radish and horseradish and the steam of turnip cabbage. The latter two were additionally pealed. Four grams of each sample were crushed with a mortar; the same plant material was used for cultivation-independent and dependent methods.

The experimental design is presented in Fig. 6: 16S rRNA gene amplicon sequencing was performed to unhide the microbial community of the different vegetables and to identify their crucial drivers. Bacteria with active myrosinase and potential biocontrol-effect, providing health-promoting properties for humans and plants, respectively, were identified by cultivation-dependent analyses. In addition, the sequences of isolates were linked with the amplicon library to explore antagonistic potential of the vegetables. The metagenomes of *E. sativa* and *B. napus* were screened for bacterial myrosinase genes in order to allocate specific functions to microhabitats.

### Illumina MiSeq data processing and analysis

For culture-independent Illumina MiSeq v2 (250 bp paired end) amplicon sequencing, the bacterial genomic DNA was extracted from 1 g of homogenized vegetable sample using FastDNA^TM^ SPIN Kit for Soil (MP Biomedicals, Solon, USA). The 16S rRNA genes were amplified in three technical replicates with universal bacterial primers 515 f and 806r^45^ including sample-specific barcodes and Illumina sequencing adaptors. Peptide nucleic acids (PNA)^46^ were used to reduce amplification of mitochondria and plastid DNA. The 30 µl of the PCR mixture contained 1× Taq&Go (MP Biomedicals, Illkirch, France), 0.25 mM of each primer, 1.5 µM PNA mix and 1 µl template DNA (96 °C, 5 min; 30 cycle of 96 °C, 1 min; 78 °C, 5 s; 54 °C, 1 min; 74 °C, 1 min; final extension at 74 °C, 10 min). Products were purified according to the protocol of Wizard SV Gel and PCR-Clean-Up System (Promega Corporation, Madison, USA). Raw sequencing data preparation, including joining forward and reverse read pairs, was carried out by LGC Genomics (Berlin, Germany). Reads were quality filtered (Phred score ≥19) over the whole sequence length using software package QIIME 1.9.1. Chimeric sequences were detected using usearch 6.1^47^ and removed. The reads were clustered to operational taxonomic units (OTUs) at 97% similarity^47^ using the UCLUST algorithm with default parameters. Taxonomic assignment of representative sequences was performed based on the reference database Greengenes release gg_13_8_99^48^. The dataset was normalized to 821 reads per sample to account a variation in the samples reaching from 821 to 92,400 sequences. The chloroplast and mitochondria sequences as well as unassigned sequences were excluded. Obviously, normalizing the dataset to 821 sequences per sample makes it hardly possible to display the whole extent of the microbiota in all samples. However, referring to literature^15,49,50^, a sampling depth of this value, or even lower, is reported to be adequate for an accurate review of alpha and beta diversity of microbial habitats.

For further data processing, taxa identified by the sequence alignments were either grouped by the vegetable type or combined corresponding to the purchase origin. Krona software package version 2.2^51^ was used to construct ring charts representing the bacterial core microbiome. Principal Coordinate Analysis (PCoA) plots were constructed to demonstrate beta-diversity by calculating the weighted UniFrac distance matrix^52^. Co-occurrence network inference tool “CoNet”, as a Cytoscape version 3.4.0^53^ add-on, was used to determine significant (p-value < 0.004) bacterial interactions. Spearman and Kendall correlation measurements, Bray Curtis and Kullback-Leibler dissimilarity matrices and the mutual information option to calculate similarity were used to ensemble interference. Benjamini-Hochberg multiple testing correlation was applied to adjust the false discovery rate. ANOSIM based on weighted unifrac distance matrix revealed which variables accounted the best for microbial differences. For functional predictions, the ‘closed-reference’ OTU database of the samples was observed using online PICRUSt Galaxy version^54^.

### Isolation of myrosinase-active bacteria and determination of enzyme activity

Myrosinase-active strains were isolated by selective enrichment in liquid M9 minimal salts media with 0.01% sinigrin (99% purity; Sigma Aldrich, St. Luis, USA) and 0.02 mM GLS crude extract (prepared according to Cools and Terry, 2012^55^; total sinigrin content of 0.045 mM as measured by High Performance Liquid Chromatography, HPLC). Solid M9-agar plates contained 0.1% sinigrin. Colonies grown on selective agar plates were genotyped using BOX-PCR fingerprinting method according to Berg *et al*.^56^. In order to amplify myrosinase related sequences, degenerated primers Myr_Fwd1 (5′-TRT GGG GYG GNG CNS TTG C-3′) and Myr_Rev2 (5′-GGR TCA ATY BSC CAG CCC CAG TC-3′) were designed based on sequence similarities of myrosinase genes *bglA* of *E. coli* O157:H7 using CLC Genomics Workbench (Aarhus, Denmark) and IDT PrimerQuest Tool (Coralville, Iowa, USA). PCR conditions were set to 95 °C, 5 min; 30 cycle of 95 °C, 30 s; 61.3 °C, 30 s, 74 °C, 40 s; final extension at 74 °C, 10 min. Myrosinase activity of cell free bacterial lysates was measured by glucose oxidase-peroxidase (GOD-POD) coupled myrosinase assay based on the protocols of Trinder (1969) and Albaser *et al*.^38,57^. HPLC system was equipped with an Ultimate3000 pump, an ASI-100 autosampler, an Ultimate3000 column oven, and an UVD 340U photodiode array detector (Dionex, California, USA). Sinigrin was separated on a Platinum (C 18) Eurostar 100 A column (150 × 4.6 mm, 5 μm, Phenomenex, Aschaffenburg, Germany), thermostated at 25 °C. The flow rate was kept constant at 0.5 ml min^−1^.

Protein-encoding nucleotide queries of putative bacterial myrosinase genes *bglA* and *ascB* were compared to metagenomics databases of *E. sativa* phyllosphere, rhizosphere and corresponding bulk soil (unpublished data), as well as four *B. napus* rhizosphere samples and two corresponding bulk soil samples (MGRast accessions: mgm4574757.3 - mgm4574760.3 and MGRast accessions: mgm4574761.3 - mgm4574762.3, respectively) using BLASTx (E-value cut-off ≤10^−18^). Access to the non-published metagenomes was exclusively granted for this study. Recovered hits were subsequently aligned (BLASTx) against nonredundant NCBI protein database to verify sequence similarity to 6-phospho-β-glucosidase.

### Screening for bacterial strains with biological control activity

Forty colonies from each vegetable sample were tested for antagonistic properties towards *V. longisporum* ELV25 Stark by a dual-culture *in vitro* assay^58^. Bio-priming on surface sterilized seeds of *B. napus* l. partim, ‘Traviata H 605886’ (KWS Saat Einbeck, Germany) was performed under gnotobiotic conditions in sterile germination pouches^59^. Sanger Sequencing results for bacterial strains were obtained from LGC Genomics (Berlin, Germany) and identified using the NCBI BLAST alignment tool. Bio-primed *B. napus* tissues were observed by fluorescent *in situ* hybridization in combination with confocal laser scanning microscopy (FISH/CLSM) using a Leica TCS SPE confocal laser scanning microscope (Leica Microsystems, Mannheim, Germany) with oil immersion objective lenses Leica ACS APO 40.0 x oil CS and Leica ACS APO 63 x oil CS. Plant tissues were fixed with 4% paraformaldehyde and 1x phosphate-buffered saline (3:1) for 6 h at 4 °C. The in-tube FISH was performed according to Cardinale *et al*.^33^. FISH probes GAM42a-Cy5 and GAM42-competitor^60^, an equimolar mixture of Cy3-labelled EUB338, EUB338-II and EUB338-III probes^61,62^ as well as NONEUB-Cy5 and NONEUB-Cy3^63^ were used to visualize colonization patterns (max. extinction/emission in nm: Cy3 548/562 and Cy5 650/670). Host structures were visualized by Calcofluor-white staining (Sigma Aldrich, St. Luis, USA) using a stationary laser at 405 nm wavelength. Maximum projections of optical z-stack slices generated micrographs of the bacterial colonization.

### Statistical analysis

Statistical analyses were performed using IBM SPSS program (version 23.0, IBM Corporation, Armonk, NY, USA). Unless otherwise stated, the p-value < 0.05 was considered to be significant. Normal distribution of the data was estimated by Shapiro-Wilk test. Differences between seedling weights were calculated by *t*-test for independent samples. Mean zones of *Verticillium* inhibition were compared by one-way ANOVA Tukey post-hoc HSD. Significant differences in microbial abundance and taxon diversity between traditionally and industrially processed vegetables were assessed using non-parametric Mann-Whitney-U-test and Kruskal-Wallis test.

### Data availability

The datasets containing the unrarefied OTU table of all samples analyzed in this study are available in the ENA European Nucleotide Archive (ENA) under the study accession number PRJEB20347, Sample accessions ERS1655946 to ERS1656001.

## Electronic supplementary material

Supplementary Information
