# Supplementary material for: Harnessing the microbiomes of Brassica vegetables for health issues
Source: Sci Rep. 2017 Dec 15;7:17649. doi: 10.1038/s41598-017-17949-z (PMC5732279; doi:10.1038/s41598-017-17949-z)
Supplement: Supplementary file 1 — Supplementary Information [file 41598_2017_17949_MOESM1_ESM.doc]

**Supplementary information**

**Harnessing the microbiomes of *Brassica* vegetables for health issues**

**Birgit Wassermann, Daria Rybakova, Christina Müller & Gabriele Berg**

Graz University of Technology, Institute of Environmental Biotechnology, Petersgasse 12, 8010 Graz, Austria

**Supplementary Table S1:** Percentage of shared OTUs between the purchase equivalents of each vegetable type.

| *Brassica* type | Shared OTUs of TP and IP equivalent (%) |
| --- | --- |
| Broccoli | 56.5 |
| Cauliflower | 60.0 |
| Horseradish | 64.7 |
| Radish | 60.0 |
| Arugula | 63.4 |
| Turnip cabbage | 59.2 |
| White cabbage | 56.2 |

**
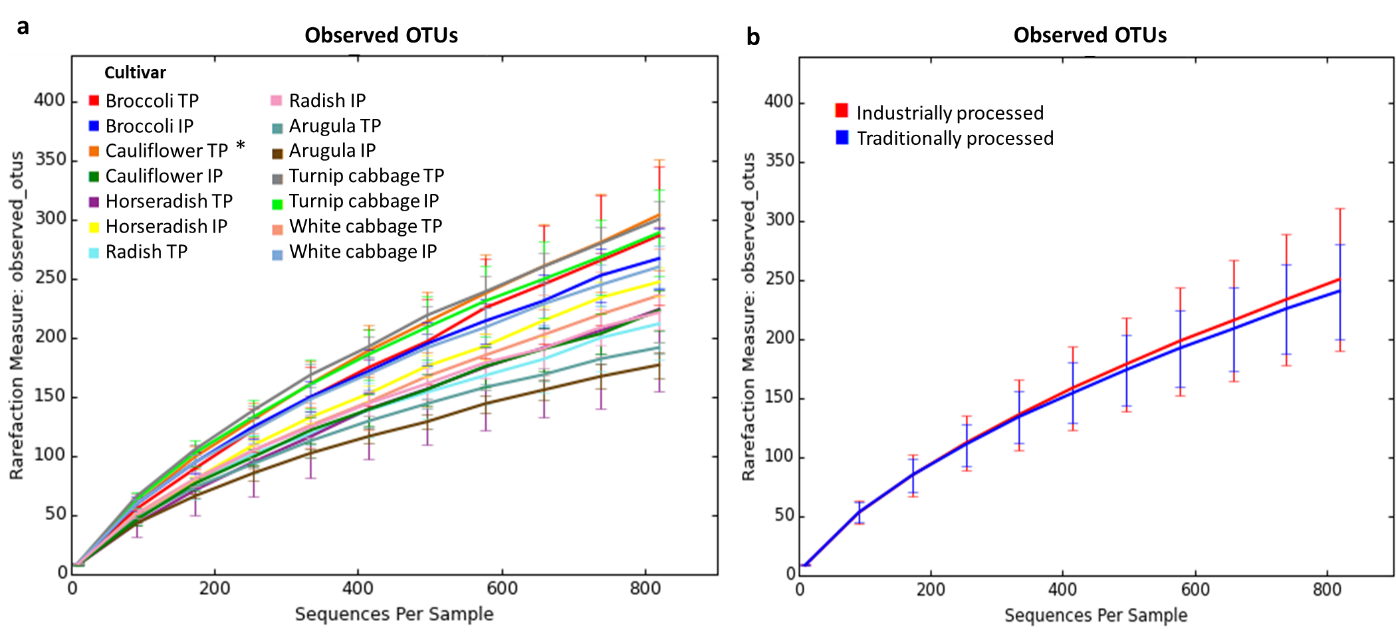
**

**Supplementary Figure** **S1**

**Alpha-diversity analysis of the microbiota inhabiting the vegetable samples investigated.** OTU table was rarefied to 821 sequences per sample. (A) Alpha-diversity of the 14 vegetable samples, indicated by the color of the curve, as described in the legend. The asterisk denotes significant difference between purchase equivalents of a vegetable type according to Shannon diversity index. (B) Pooled sample of all traditionally processed vegetables (red line) compared to the pooled industrially processed sample (blue line).

**Supplementary Table S2: OTU richness of *Brassica*** samples in the rarefied OTU table.

| Cultivar | Shannon* |
| --- | --- |
| Broccoli TP | 7.3 ± 0.5^abc^ |
| Broccoli IP | 7.2 ± 0.3^bc^ |
| Cauliflower TP | **7.5 ± 0.4^c^** |
| Cauliflower IP | **6.3 ± 0.4^ab^** |
| Horseradish TP | 6.0 ± 0.9^abc^ |
| Horseradish IP | 6.7 ± 0.0^ab^ |
| Radish TP | 6.7 ± 0.7^bc^ |
| Radish IP | 6.9 ± 0.2^abc^ |
| Arugula TP | 6.7 ± 0.1^bc^ |
| Arugula IP | 6.3 ± 0.1^bc^ |
| Turnip cabbage TP | 7.7 ± 0.2^ab^ |
| Turnip cabbage IP | 7.4 ± 0.5^abc^ |
| White cabbage TP | 6.6 ± 0.4^abc^ |
| White cabbage IP | 7.5 ± 0.2^a^ |

*The common letters following the mean values and standard deviations (mean ± SD) indicate a non-significant difference for each strain (p<0.05) according to ANOVA post-hoc Tukey-HSD T-test. Significant differences between purchase equivalents of a vegetable type are highlighted in *bold*.


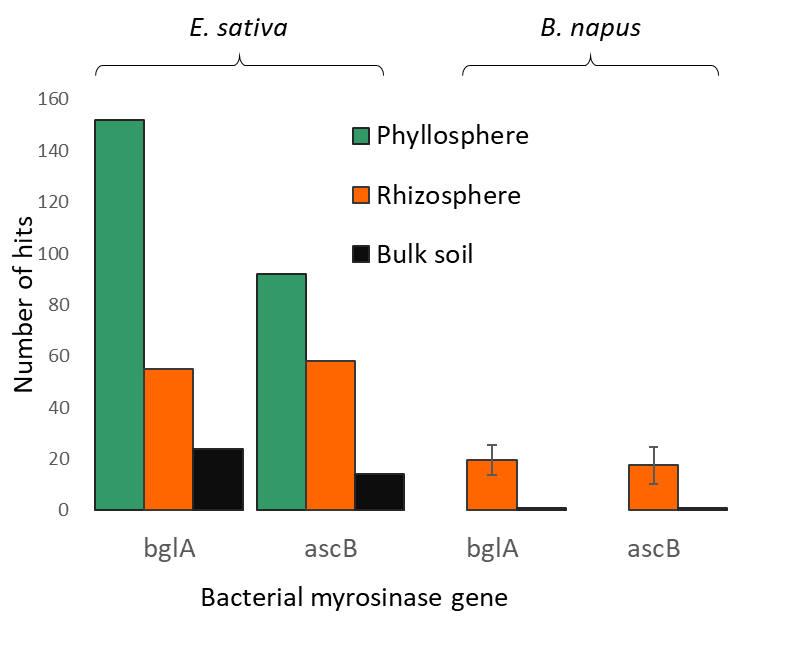


**Supplementary** **Figure S2**

**Abundance for bacterial myrosinase genes in the metagenome of *E. sativa* and *B. napus*.** No phyllosphere sample was available for *B. napus*.

**
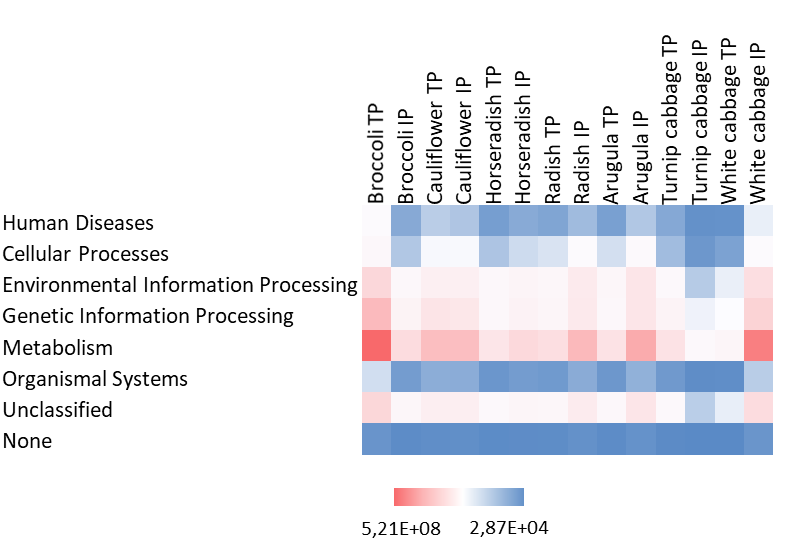
**

**Supplementary** **Figure S3**

**PICRUSt analysis representing assignment of vegetable-associated microbiota to specific functions.** Functional diversity was annotated to KEGG level 1 orthologies representing the functional composition of the purchase equivalents of each vegetable type (left part of the heatmap), as well as the samples pooled together according to their processing pathway (right part of the heatmap). The heatmap scales from 5.21E+08 to 2.87E+04 sequence hits.
